# Effects of BPA and BPS exposure limited to early embryogenesis persist to impair non-associative learning in adults

**DOI:** 10.1186/s12993-015-0071-y

**Published:** 2015-09-17

**Authors:** Mahlet D. Mersha, Bansri M. Patel, Dipen Patel, Brittany N. Richardson, Harbinder S. Dhillon

**Affiliations:** Department of Biological Sciences, Delaware State University, Dover, DE 19901 USA; Sussex Tech High School, Georgetown, DE 19947 USA; Neuroscience and Psychology Program, University of Pittsburgh, Pittsburgh, PA 15260 USA

**Keywords:** Bisphenol-A, Bisphenol-S, Endocrine disrupting compounds, Habituation, *C. elegans*, Embryonic exposure, Development, Autism, BPA, BPS, EDC

## Abstract

**Background:**

Bisphenol-A (BPA) is a polymerizing agent used in plastic bottles and several routinely used consumer items. It is classified among endocrine disrupting chemicals suspected to cause adverse health effects in mammals ranging from infertility and cancer to behavioral disorders. Work with the invertebrate lab model *Caenorhabditis elegans* has shown that BPA affects germ cells by disrupting double-stranded DNA break repair mechanisms. The current study utilizes this model organism to provide insight into low-dose and long-term behavioral effects of BPA and bisphenol-S (BPS), a supposed safer replacement for BPA.

**Findings:**

Experiments presented in our report demonstrate that the effects of embryonic exposure to considerably low levels of BPA persist into adulthood, affecting neural functionality as assayed by measuring habituation to mechano-sensory stimuli in *C. elegans.* These results are noteworthy in that they are based on low-dose exposures, following the rationale that subtler effects that may not be morphologically apparent are likely to be discernible through behavioral changes. In addition, we report that embryonic exposure to BPS follows a pattern similar to BPA.

**Conclusions:**

Building upon previous observations using the *C. elegans* model, we have shown that exposure of embryos to BPA and BPS affects their behavior as adults. These long-term effects are in line with recommended alternate low-dose chemical safety testing approaches. Our observation that the effects of BPS are similar to BPA is not unexpected, considering their structural similarity. This, to our knowledge, is the first reported behavioral study on low-dose toxicity of any endocrine disrupting chemical in *C. elegans*.

## Background

The human health impact of bisphenol-A (BPA or 4,4′-isopropylidenediphenol), which is in commercial use since the 1960s, has been under scrutiny in recent years. BPA is used in the polymerization process of polycarbonate plastics and resins as well as in the manufacture of commonly used products ranging from thermal paper used for sales receipts to flame retardant precursors, dental sealants, and the inside coating of beverage and food cans including those used for infant formula [1, 2]. Considering its widespread use, it is not surprising that 90 % of Americans have traceable amounts of BPA in their urine [3]. BPA is suspected to induce pre-term birth in pregnant women [4], and is reported to cause adverse health effects including nervous system disorders in both, children and adults [5, 6].

BPA exhibits hormone-like properties, mimicking 17-β Estradiol (E_2_) and is classified as an endocrine disrupting compound (EDC) [2]. E_2_ is known to act through different members of the estrogen receptor family including ER-α, ER-β and ERR-δ, which play critical roles in the regulation of embryonic development including neuronal survival and plasticity [7]. Considering the fundamentally essential roles of E_2_ in development and the EDC properties of BPA, a number of recent studies have focused on the biological effects of exposure to BPA. Exposure to BPA affects nervous system function with chronic exposure leading to an increase in dopamine D_1_ receptor expression in mouse limbic forebrain, which can result in hyperactivity, attention deficits and heightened sensitivity to drugs of abuse [8, 9]. Furthermore, in mouse embryos exposed to BPA, the long-term neuronal defects that persevere into adulthood have been shown to be epigenetically mediated through DNA methylation [10].

**Fig. 1:**
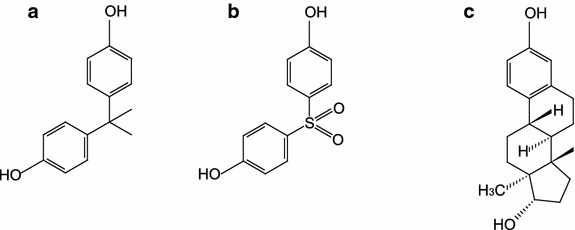
Chemical structures of **a** BPA, **b** BPS and **c** estradiol. Key structural similarities with the naturally occurring steroid hormone 17-β estradiol may underlie observed endocrine disrupting properties of BPA and BPS

With the trickle of reports incriminating BPA in contributing to adverse health effects and a considerable increase in public awareness, some industries in the United States have initiated self-regulatory measures towards voluntary replacement of BPA with a purported safer substitute bisphenol-S (BPS or 4,4′-sulfonlydiphenol) which shares remarkable structural similarity to BPA and estradiol (Fig. 1). Recent studies have shown that BPS has comparable anti-androgenic effects and is likely to regulate estrogenic transcription at a level comparable to estrogen itself [11, 12].

Considering the conserved nature of genes of *Caenorhabditis elegans* with mammals, including its steroid hormone-receptor genes [13], researchers have begun utilizing this genetically tractable lab model to understand the effects of EDCs to obtain foundational insight on the mechanisms of BPA action [14, 15]. A key report has linked increased sterility and embryonic lethal effects of BPA to genomic instability caused due to breakdown of double-stranded DNA break repair mechanisms [14, 15]. However, it is important to note that this study is based on internal BPA concentrations at par with those used in commonly used mammalian models equivalent to occupational exposure levels of 2 ppm [14, 15]. Newer and alternate approaches to chemical safety determination indicate that low doses of toxic chemicals are associated with distinct pathologies and that the observations at high doses may not necessarily predict low-dose toxicity [2, 16]. A low-dose, based on US Environmental Protection Agency and US National Toxicology Program panel guidelines, [16] may be defined as any dose below the level of one which has been reported to cause an observable biological change or damage [2]. Our focus is on studying the low-dose effects of BPA on the functional integrity of the nervous system. Although a diverse range of behavioral effects attribute to BPA exposure have been studied in mammalian models [6], its behavioral effects have not been studied in *C. elegans*, a model with the potential to unravel the developmental basis of the observed behavioral anamolies.

To follow up on the study demonstrating increased embryonic lethality and genomic instability caused by BPA [15], we have assessed the low-dose effects on adult behavior as a function of embryonic exposure. Our working hypothesis states that low-dose exposure to BPA and BPS during development leads to quantifiable neural dysfunction, as opposed to morphological abnormalities or gross phenotypes observed at higher doses. We chose to focus on habituation behavior, which is a form of non-associative learning characterized by reduced response to a repeated stimulus [17]. In humans, habituation is a well-documented neural endo-phenotype of several complex behavioral disorders including schizophrenia and autism [18, 19]. The mechanistic correlates of habituation have remained remarkably conserved from simple invertebrates to mammals indicating its essential role in nervous system function [17]. To measure the developmental effects of BPA and BPS, we tracked the behavior of *C. elegans* exposed to these compounds in their embryonic period of life. We have examined the effects of low-dose exposure of BPA on fecundity and behavior, followed by testing whether BPS is truly a safe alternative. Our results establish that low-dose embryonic exposure to both BPA and BPS can trigger long-term effects on the surviving adults’ neuronal function as assayed through habituation behavior.

### Adult animals exposed to BPA as embryos have decreased progeny

**Fig. 2:**
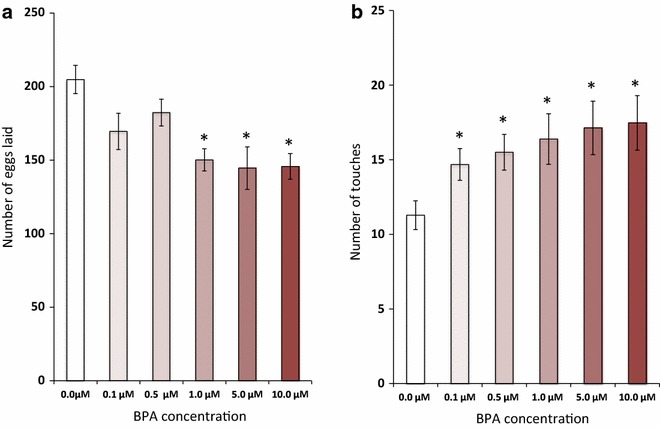
BPA affects fecundity and neural functions. **a** Embryos exposed to BPA (1 μM and higher concentrations) laid significantly fewer eggs as adults, as compared to controls (*bars* depict the mean number of eggs laid; n = 10, *p < 0.05). **b** Adults arising from embryos that had been exposed to BPA required higher number of stimuli in order to habituate to touch (*bars* represent the mean number of gentle taps required for habituation; n = 60, *p  <  0.05). *Error bars* denote SEM. Statistical analyses were done using one-way ANOVA followed by Tukey’s post hoc analysis

BPA (obtained from Sigma-Aldrich, St. Louis, MO) was solubilized in 10 % ethanol to make a 100 μM stock solution and subsequent dilutions were made in S-buffer (0.1 M sodium chloride 0.05 M potassium phosphate, pH 6.0). *C. elegans* embryos were isolated from gravid adults using basic hypochlorite solution [20] and exposed to 0.1–10 μM BPA concentrations. After 4 h of BPA exposure, the embryos were transferred to nematode growth media (NGM) plates seeded with *E.coli* OP50 (without exogenous BPA) and incubated at 20 °C. Upon reaching the L3 larval stage, individual worms were transferred to fresh, seeded plates and the total numbers of eggs laid by each individual adult were counted. We observed a statistically significant decrease in the number of eggs laid at BPA concentrations of 1.0 μM and higher (Fig. 2a). A dramatic decrease in the number of eggs laid by *C. elegans* that were continually exposed to higher BPA concentrations (≥1 mM) beginning from the embryonic period and continued throughout adulthood, has been reported previously [15]. Our observations are based on exposure to lower doses that were limited to the embryonic period.

### Embryonic BPA exposure slows the habituation response of adult worms

Next, we used anterior touch sensory functional plasticity to examine the habituation behavior of adult worms [21] exposed to BPA in their embryonic phase. BPA exposure of embryos was performed as described above, and the exposed embryos were transferred to seeded NGM plates without exogenous BPA. Well-fed 3 day old adult worms were assayed for habituation to anterior touch as described previously [21]. Briefly, animals were given repeated gentle anterior touch stimuli with 10 s inter-stimulus-intervals until they no longer responded to the stimulus. The number of stimuli required by an animal until it no longer moved backward was recorded. We found that worms exposed to BPA at even the lowest concentration tested (0.1 μM) required more stimuli to become habituated, when compared to worms exposed to vehicle alone (Fig. 2b).

### Exposure to BPS causes effects similar to BPA

**Fig. 3:**
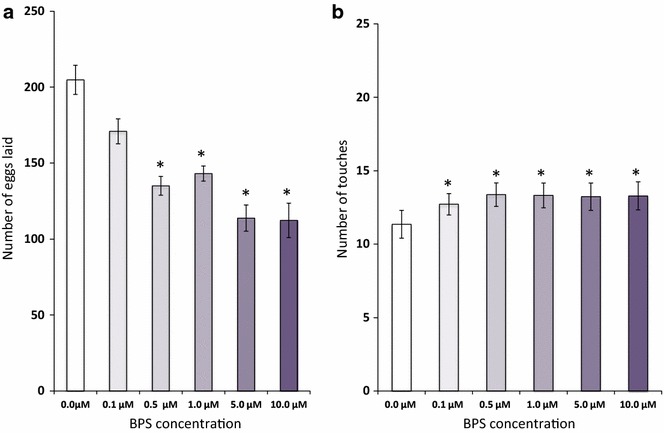
BPS exerts effects similar to BPA. **a** Embryos exposed to BPS (0.5 μM and higher concentrations) laid significantly fewer eggs as adults, as compared to controls (*bars* depict the mean number of eggs laid; n = 10, *p < 0.05). **b** Embryonic exposure to BPS results in slower habituation (*bars* represent number of gentle taps required for habituation; n = 60, *p  <  0.05). *Error bars* denote SEM. Analysis done with one-way ANOVA followed by Tukey’s post hoc analysis

The above results with low-dose BPA led us to carry out a similar set of experiments with BPS (Sigma-Aldrich, St. Louis, MO). Exposure to BPS, egg count and habituation measurements were carried out using essentially identical protocols as described for BPA above. As in the case of BPA, we found that exposure to BPS led to a significant decrease in the number of eggs laid at 0.5 μM and higher concentrations (Fig. 3a). Additionally, adult worms, which developed from surviving embryos that were exposed to BPS (ranging from 0.1 to 10 μM) displayed a decrease in habituation when compared to animals exposed to vehicle alone (Fig. 3b). We did not observe any morphological differences in our exposed embryos or adults for either BPA or BPS, conceivably due to our use of considerably lower concentration than those used in a previous *C. elegans* study that was based on continual exposure to high levels of BPA [15].

## Discussion

Our results demonstrate that in *C. elegans* the effects of embryonic exposure to considerably low levels of BPA persist into adulthood, affecting their neural function as assayed by measuring their habituation to anterior touch sensory stimuli. Additionally, we found that BPS, intended to be a safer alternative to BPA, also caused decreased habituation suggesting that it is likely to exert its action in a similar manner as BPA. While confirming previously reported decreased egg-laying caused by continual exposure of *C. elegans* to BPA at ≥1 mM concentrations [15], our results extend these observations by demonstrating decreased fecundity at significantly lower concentrations (as low as 1 μM BPA and 0.5 μM BPS) with exposure limited solely to the embryonic period. Due to their hormone-like properties and structural similarity with estradiol, BPA and BPS may have the potential to interfere with estradiol’s modulatory role in synaptic plasticity [22]. It is notable that mammalian studies have shown that BPA exposure can increase levels of dopamine in the midbrain [23] as well as up-regulate dopamine D_1_ receptor expression [8]. Interestingly, postulated mechanisms of mechano-sensory habituation in *C. elegans* point to a central role for dopamine [17, 21, 24, 25]. Considering the above reports, along with the results presented here, future studies on the effects of BPA and BPS on dopamine regulation may yield valuable information on the mechanisms by which these EDCs affect neuronal function. In conclusion, our study extends knowledge gained from previous reports by examining low-dose exposure in the *C. elegans* model by utilizing an evolutionarily conserved behavior as a surrogate for integrity of neural function. Extending the assay used with our model has the potential to uncover subtle behavioral effects of low-dose exposure to suspected neurotoxic compounds that may not cause phenotypically visible abnormalities.

## Availability of supporting data

The data sets supporting the results of this article are included within this article and its figures.

## Abbreviations

BPA: bisphenol-A
BPS: bisphenol-S
EDC: endocrine disrupting compound
NGM: nematode growth media
